# Silicon and germanium nanocrystals: properties and characterization

**DOI:** 10.3762/bjnano.5.189

**Published:** 2014-10-16

**Authors:** Ivana Capan, Alexandra Carvalho, José Coutinho

**Affiliations:** 1 Rudjer Boskovic Institute, P.O. Box 180, 10000 Zagreb, Croatia; 2 Graphene Research Centre and Department of Physics, National University of Singapore, 117542, Singapore; 3 Department of Physics and I3N, Campus Universitário de Santiago, 3810-193 Aveiro, Portugal

**Keywords:** characterization, germanium, modelling, nanocrystals, silicon

## Abstract

Group-IV nanocrystals have emerged as a promising group of materials that extends the realm of application of bulk diamond, silicon, germanium and related materials beyond their traditional boundaries. Over the last two decades of research, their potential for application in areas such as optoelectronic applications and memory devices has been progressively unraveled. Nevertheless, new challenges with no parallel in the respective bulk material counterparts have arisen. In this review, we consider what has been achieved and what are the current limitations with regard to growth, characterization and modeling of silicon and germanium nanocrystals and related materials.

## Review

### I Introduction

Nanocrystals (NCs) have emerged as one of the preferred ways to control quantum phenomena at the nanoscale. This control leads to spectacular properties and to a wide field of applications ranging from memory devices, solar cells and thermoelectrics to light emitters, spintronic devices and printable electronics.

The use of semiconductor NCs instead of two-dimensional floating gate devices such as metal oxide semiconductor (MOS) field effect transistors and memory devices makes memory less sensitive to common problems such as leakage current and dielectric breakdown and allows for an ultimate miniaturization without electrical instabilities [1]. Moreover, nanostructures offer significant breakthroughs in photovoltaics. It is believed that quantum dot (QD) solar cells have the potential to reach a maximum conversion efficiency of about 66% [2]. A colloidal nanocrystal solar cell, which combines all the advantages of organics (scalable and controllable synthesis) with transport properties comparable to traditional photovoltaic semiconductors, has been demonstrated [3]. Different approaches have been made to achieve light emission from group-IV semiconductor nanostructures despite the indirect nature of the energy gaps. The quantum confinement of carriers has led to efficient luminescence and electroluminescence of semiconductor nanostructures, mostly Si and Ge NCs embedded in an oxide matrix [4].

Theoretical research on NCs was also triggered by the interest in their optical properties [5–7], namely following the work of Furukawa and Miyasato with Si NCs [8], which reported the opening of the optical gap of small Si QD by more than 100% with respect to bulk Si. Within a "particle-in-a-box" description, the effect was simply cast as a power law *E*_gap_ = *E*_bulk_ + α/*R^n^* [9], where *R* is the particle size, α is a confinement factor and *n* is usually 1 < *n* < 2, depending mostly on the surface termination and host material (for a review see [10]). Despite its popularity, such an empirical approach overlooks important physical aspects such as the chemical nature of the NCs or the dielectric mismatch across their boundaries [11].

One important issue that has led to considerable debate relates to the screening effects within the NCs [12–15]. An accurate theory for the dielectric screening is essential to understand the response of nanostructures to an external electro-magnetic field, or the localization of dopant states and excitations. There is consensus now that even for NCs with a diameter as small as 2 nm, the spatially averaged electronic screening within the core is virtually identical to that in bulk, decreasing to the vacuum value close to the polarized surface [16]. Confinement due to underscreening is essentially a surface/interface effect that manifests itself when the surface-to-volume ratio of the structures becomes large [14].

Many important questions related to impurities and surface chemistry have to be answered by using quantum-chemical models. With the development of efficient linear-scaling density functional methods along with the steady drop of CPU-time costs, we are now able to solve by first-principles the all-electron problem of systems with a few thousands of atoms [17]. This has led to the understanding of more complex problems such as doping [18–20], and electronic transport across NC solids [21–22]. These are among the advances that will be reviewed below.

### II Growth and characterization

Much of current research on Si/Ge NCs is focused on the preparation and characterization of NCs embedded in a SiO_2_ matrix. In this paper, we have restricted our analysis to growth techniques, such as magnetron co-sputtering and ion implantation. With these techniques, it is possible to produce and to control the space-/size-distribution and ordering of NCs. Both benefits and shortcomes of these techniques will be pointed out along the text. It should be noted that other important and wide-spread techniques are also used for the growth of Si and Ge NCs, namely PECVD (CVD) and MBE. From the experimental point of view, the synthesis of free-standing NCs will not be covered within this review. However, those issues are visited in Section III of this paper, which deals with the first principles modelling of the NCs.

#### II.1 Magnetron co-sputtering

This is basically a technique for thin film deposition. Together with thermal evaporation and pulsed laser deposition (PLD), they are all fabrication methods based on the production of sub-stoichiometric oxides, with thermal evaporation being the simplest among them. The NCs size, distribution and shape can be controlled by varying sputtering time, composition of sputtered material, as well as the annealing temperature, time and atmosphere. Since NCs embedded in the oxide matrix have a large interface-area-to-volume ratio, they show a high density of interface defects [23]. In such cases, the role of the interface and interface-related traps cannot be neglected.

The interface traps are located at the substrate/oxide-matrix interface (most commonly Si–SiO_2_) and at the NCs/oxide-matrix interface. Unlike the other traps located in the oxide matrix (fixed and mobile oxide charges), interface traps are in electrical communication with the underlying substrate, and therefore largely affect the electrical properties of such structures [24]. Passivation of deep levels caused by the interface is necessary before NC-based optoelectronic devices can become reality. Vacuum annealing, which is used for NC crystallization, is known to increase the density of the interface traps. However, most of the interface traps can be neutralized by low-temperature (about 450 °C) annealing with hydrogen or forming gas [24].

In general, the formation of Ge NC is much more complicated than that of Si NC because GeO_2_ is thermodynamically less stable than SiO_2_, which leads to a higher concentration of defect states [23]. Moreover, the high difference between the thermal expansion coefficients of Ge and SiO_2_ and the large lattice constant of Ge results in mechanical stress in the system [25]. However, due to the lower binding energy of Ge atoms in comparison with Si atoms, Ge NCs can be formed in samples annealed at significantly lower temperatures (600–900 °C).

Martin-Sánchez et al. [26] have reported that a crystallization of as-deposited amorphous Ge NCs can be achieved by a short treatment at relatively low temperatures, which significantly decreases the thermal budget, when compared to other growth techniques. Moreover, it implies that PLD should be considered as an excellent alternative to the widely used magnetron co-sputtering technique for the deposition of complex oxide thin films and NCs.

#### II.2 Ion implantation

An ion beam stands as a highly versatile tool for a variety of purposes including synthesis, modification and characterization of materials. The synthesis of NCs made of silicon and germanium by means of ion beams has been extensively studied in the past years [23]. QDs size, shape and distribution can be controlled by varying the implantation conditions (implantation energy and dose) and subsequent annealing. It is a well-known fact that Si ion implantation of SiO_2_ is characterized by the production of a large number of oxygen vacancies and other defects in the oxide matrix. These defects enhance the QD formation and the formation of the sub-oxide interface states [23].

As ion implantation is unavoidable for CMOS technology today, it is desirable to use it not only for the fabrication of NCs but for the doping of NCs as well. Over the past decade, phosphorus-and erbium-doped Si NCs have attracted a great deal of attention, due to the promising optoelectronic applications [27]. It has been shown that in order to study the donor doping and dependences of the PL intensity in such systems, it is crucial to understand and control the presence of defects. The effects of donors and defects to the PL of Si NCs are strongly overlapping [28]. Moreover, it was reported that the density of interface-related defects may be decreased by light phosphorus doping [29].

An alternative method to the doping of NCs by using ion implantation is by means of neutron transmutation doping (NTD). NTD is a technique commonly used to dope bulk semiconductors, but for NCs is not much explored. Recently, the promising application of NTD for arsenic-doping of Ge NCs has been reported [30].

#### II.3 Properties and characterization

To obtain all necessary information regarding the properties of NCs, it is desirable to perform a complete characterization that includes structural, optical and electrical specifications. Most characterization reports on semiconductor NCs start with transmission electron microscopy (TEM) data. This is a widespread and well-known technique, which gives extremely valuable structural information. TEM has been used to study Si NCs produced by reactive evaporation in one of the first approaches to produce embedded semiconductor NCs, the so-called superlattice approach [31]. This method enables an easy and well-defined control over the particle size, density and ordering.

Together with TEM, there are other useful techniques that can provide information regarding the structural properties of NCs. One of them is grazing incidence small X-ray scattering (GISAXS), which is a non-destructive technique for the structural characterization of NCs supported on a substrate [32] and NCs embedded in a matrix [33]. From the GISAXS analysis, it is possible to determine size, shape, inter-NC distance and size distribution of NCs.

**Figure 1:**
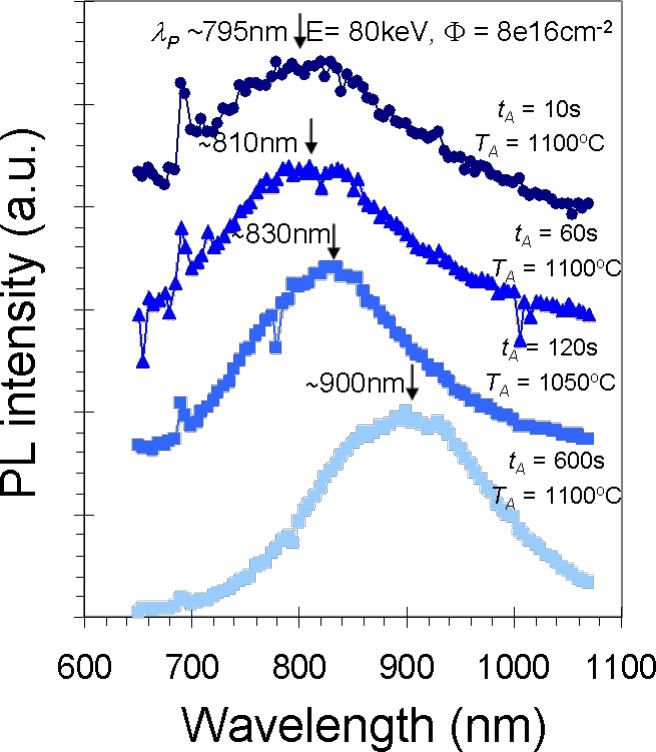
Evolution of the Si NC photoluminescence spectra (offseted for clarity) with high temperature annealing (*t*_A_ = time, *T*_A_ = temperature) for a thin (500 nm) oxide (on sapphire) implanted with Si^+^ at *E* = 80 keV to a dose of 8 × 10^16^ cm^−2^) [34].

Among all properties of semiconductor NCs, the optical properties have been the most extensively studied, usually by using photoluminescence (PL). Si NCs often show strong luminescence intensity in the visible and near-infrared region. A size-dependent blue shift of the luminescence comparable to porous Si is well documented. Figure 1 shows PL spectra of Si NCs produced by ion implantation and subsequent annealing [34]. The emission is tuned by varying the implantation and annealing conditions (yielding control over of the NC size).

Interaction of Si NCs with Er ions has attracted a special attention due to the possibility of producing optoelectronic devices operating in the telecommunications band around 1.5 μm. Recent studies have shown that doping with phosphorus can result in either a quenching or an enhancement of the Si NCs PL, depending on the dopant concentration and NC sizes [28,35]. Crowe et al. [35] have shown that as phosphorus accumulates at the nanocrystal oxide interface it leads to the passivation of the dangling bonds as observed by the luminescence enhancement. This is only valid for small NCs, while the enhancement diminishes as the density of larger NCs increases.

As already mentioned, one of the main applications of the embedded NCs is in memory devices. To check the charge trapping properties of the embedded NCs, different electrical characterization techniques could be applied. The most common is the capacitance-voltage (*C*–*V*) technique that gives information about the memory window. Moreover, *C*–*V* gives valuable information as far as interface-defects and oxide defects are concerned. There are numerous studies on the charge-trapping properties of embedded NCs. However, it should be noted that in a significant number of those, the effects of defects (either interface-related or in the oxide) have been completely neglected, and the observed memory window (the flat band voltage shift Δ*V*_FB_) has been unconditionally ascribed to the NCs trapping properties. As some studies indicate [1,26] these issues should be seriously considered.

**Figure 2:**
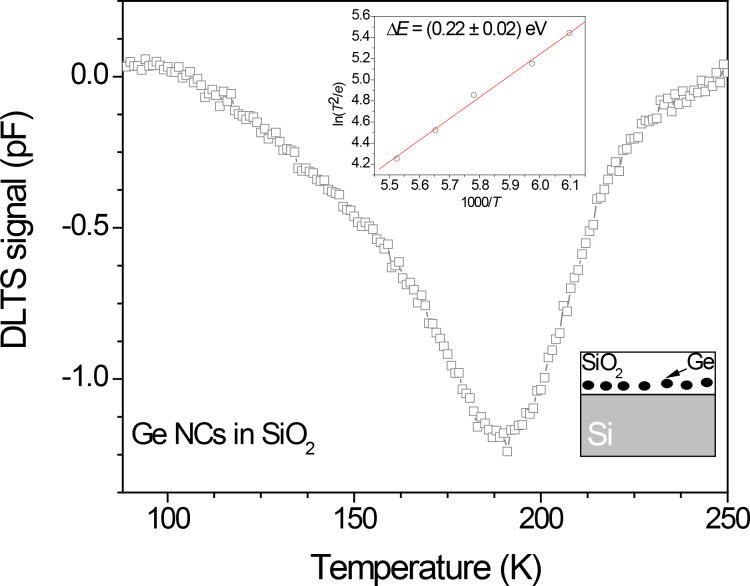
DLTS spectrum of a MOS structure with Ge NCs embedded in the oxide, with Arrhenius plot in the inset. By careful adjustments of the measurement settings, the thermal emission of electrons from the NCs is detected by DLTS (for more explanations on DLTS analysis see [24,36–38]).

To obtain even more information about defects in embedded NCs and at their interfaces, a capacitance transient measurement (through deep-level transient spectroscopy, DLTS) could be applied. DLTS has been mostly used for studying defects in semiconductors [36]. It has been successfully applied in studying the Si–SiO_2_ interface related defects [37] and in studying NCs [25,38]. In the case of NCs, the DLTS can detect only charge carriers thermally emitted from the NCs. Figure 2 shows a typical DLTS spectrum of the simple MOS structure, with Ge NCs embedded in the SiO_2_ film deposited on a Si substrate. In order to check for the existence of the deep level traps coming from the Si/SiO_2_ interface, temperature dependent *C*–*V* measurements have been performed. The activation energy of the electron emission has been determined from the Arrhenius plot (given in the inset). The observed deep level trap at 0.22 eV resembles those already reported [25,38] and it has been already ascribed to charge trapping centers associated with Ge NCs.

Another open and still unresolved problem is the electrical transport across doped NC films. Here, significant progress has been made in free-standing Si NC films [22], but much more effort is needed, for instance by using different electrical characterization techniques, to understand the electrical properties of doped and embedded NCs.

### III Electronic structure models

Effective mass models and the more elaborate k·p perturbation theory have been used to give insight into the quantum confinement [39], dispersion, as well as on the dependence of the electronic levels of nanocrystals and quantum dots on strain and electric fields [40]. Such results are general and have been helpful to understand the trends found by experimental and atomistic modeling studies. More recently, significant understanding of the relationships between structure, chemistry and electronic structure has been obtained from first-principles calculations based on density functional theory. From a theoretical perspective, group-IV nanocrystals are usually divided into free-standing and embedded. The first class of models mimics the NCs grown, for example, by using plasma methods; the second class comprises nanocrystals embedded in another material, as for example Si nanodots in SiO_2_, or in a different phase of the same material, for example crystalline Si nanodots in amorphous Si.

Free-standing nanocrystals in vacuum can be modeled by using real-space boundary conditions, i.e., requiring the wavefunction to vanish far from the nanocrystal. An alternative approach is to impose periodic boundary conditions (“particle in a box”). This has the advantage of allowing for expansion of the charge density in plane-waves and the use of numerical approaches developed for crystalline solids. However, care has to be taken to ensure that there is little interaction between periodic images of the system. This is particularly relevant when treating systems with an electric charge or dipole [41]. Embedded nanocrystals are best modeled by using periodic boundary conditions. In that case, one of the main difficulties consists of reproducing the correct structure of the interface and the respective oxidation numbers [42]. The gap between such ideal models, which provide insight into quantum effects, and real scale systems is difficult to bridge, but there have been some attempts at building multi-scale modeling frameworks [43]. Still, there is ample room for development in the modeling of growth, interaction with solvents, and other multi-scale phenomena. In this review, however, we will concentrate on quantum-chemical modeling at the nanoscale.

#### III.1 The Hamiltonian and the many-body problem

The greatest challenge associated with modeling NCs is the huge number of atoms required for the model to meet the sizes of even the smallest particles that were realized experimentally (about 1000 atoms for ca. 3 nm). Such sizes are close to the practical limits of present-day first-principles density functional theory implementations. Various full-DFT implementations by using localized basis sets such as Aimpro [17,44], Conquest [45], Onetep [46], OpenMX [47] and Siesta [48] exhibit order-*N* scaling [49] and are advertised as enabling calculations with up to *N* ≈ 10^4^ atoms. Larger systems have been treated by using tight binding or semi-empirical methods [50].

Further, localized basis sets, such as Gaussian-type orbitals, are convenient in the case of free-standing nanocrystals, both because they can be combined with real-space boundary conditions and because they result in sparse Hamiltonian and overlap matrices that can be efficiently diagonalized. Notwithstanding, many implementations of density functional theory traditionally used in solid state physics rely on the use of plane-wave basis sets for expansion of wavefunctions and charge density. A disadvantage of this approach is that to make the basis size finite it is then necessary to impose periodic boundary conditions, allowing for sufficiently large vacuum spaces in between a nanoparticle and its image. The large supercell size reflects then into a great number of plane waves, which is independent on the ratio between the volumes of matter and vacuum.

#### III.2 Theoretical prediction of observables

**III.2.1 Optical response:** The indirect band-gap nature of bulk Si has been one of the main drawbacks of this material. It has placed Si away from many optical applications such as lasers and LEDs, in favor of the more expensive and less environmental friendly III–V semiconductors. While radiative recombination on compound semiconductors is an efficient process, in Si such transitions are rather unlikely to take place as the large k**-**space mismatch between conduction band minimum and valence band maximum states implies the involvement of phonons. On the other hand, the dispersionless and tunable nature of the electronic structure of Si NCs holds many promises in all-Si optoelectronics. In this respect, theoretical modeling has provided many insights. Besides the size and shape effect to the energy and rate of optical transitions, the chemical nature of the surface, shell or host where the NC is embedded play a critical role [51]. For instance, Guerra and Ossicini [52] showed that while radiative recombination of H-saturated Si NC is strongly dependent on the size of the crystallites, in hydroxy-terminated or SiO_2_-embedded NCs the optical yield is mostly determined by the fraction of oxygen termination.

First-principles modeling has also been insightful in the study of local polarizations that take place at Si NC/SiO_2_ interfaces [53]. The abrupt change of chemical species (with different electronegativities) at the edge of the NCs induces charge displacements that can be accounted for by using pseudopotentials. It has been found that polarizations due to the oxide interface have two important effects, namely (i) to quench the low energy absorption region and (ii) a blue-shift of some particularly intense transitions.

Another important aspect is the role of defects. Si dangling bonds or radicals are strongly localized being effective traps for both electrons and holes [21]. They are therefore likely to degrade the optical yield. On the other hand, shallower states like those produced at the Si/SiO_2_ core–shell interface of oxidized Si NCs are most likely to shift the absorption/emission spectra with respect to pristine NCs [54].

Inclusion of many-body effects is important especially for small nanoparticles in which the exciton binding energy can be large [55]. These can be calculated by using the Bethe–Salpeter equation [7]. However, for nanocrystals with a radius larger than 0.6 nm, the self-energy and the Coulomb corrections almost exactly cancel each other, and one-particle calculations actually give accurate values for the excitonic gap(s).

**III.2.2 Doping of Si nanocrystals:** The deliberate introduction of dopants into materials lies at the heart of microelectronics. A prototypical example is the replacement of a few lattice sites in a billion in crystalline Si, for instance by phosphorous or boron, to confer good electrical conductivity to an otherwise poor insulator. In the same way, doping NCs will play an analogous role in future artificial solids or meta-materials made of wave-function engineered particles.

Although many promising applications of Si NCs rely on the possibility to tune their electronic states by exploring surface effects, the fact is that this is only possible in ultra-small NCs in which effective electrical doping, which leads to a spontaneous ionization of dopants at room-temperature, is yet to be demonstrated. Notwithstanding, the introduction of dopants in Si NCs has been unequivocally demonstrated, for instance by monitoring the ^31^P hyperfine splitting of the phosphorous donor state during electron paramagnetic resonance experiments [56], or from photoluminescent transitions of bound excitons to boron [57].

In small NCs, in which surface conditions affect the electronic states, theory predicts that phosphorous and boron levels are rather deep with carrier binding energies of the order of 1 eV, anticipating serious difficulties in finding suitable dopants to operate at room temperature [21,58–60].

**Figure 3:**
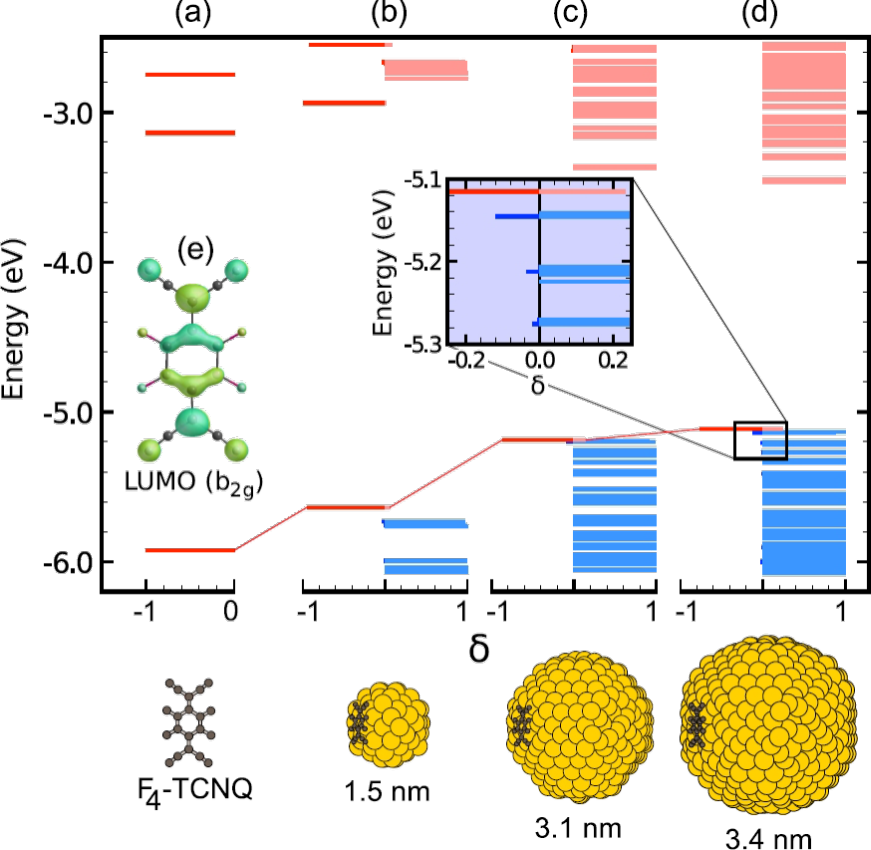
Hybridization between F_4_-TCNQ and Si NC one-electron states. (a) Isosurface plot of the F_4_-TCNQ lowest unoccupied state (b_2g_ symmetry), and (b–e) Kohn–Sham eigenvalue energy diagrams. The energy-level diagrams are for (a) isolated F_4_-TCNQ and (b–e) for F_4_-TCNQ adsorbed on Si NCs of increasing size. Each state is represented by a bar of unitary length, with the abscissa of the left- and right-hand ends of the bar indicating, respectively, the relative localization on the molecule (negative values) and on the NC (positive values). Reprinted with permission from [20]. Copyright 2011 The American Physical Society.

In this respect other routes for doping have been under investigation. Namely, transfer doping by means of molecules with high electron affinity or low ionization potential stands as a rather promising alternative. For instance, Carvalho et al. [20] suggested that the F_4_-TCNQ (7,7,8,8-tetracyano-2,3,5,6-tetrafluoroquinodimethane) molecule, which is commonly used as a p-dopant in organic electronics, could be a candidate to produce holes in the Si NCs, as shown in Figure 3. They also showed that about three to four molecules adsorbed to the NC surface are able to produce a positively charged NC, although the first excitation of the doped system was almost 1 eV, again indicating that free-holes are unlikely to be produced at room temperature. These results were confirmed recently by means of electrical measurements combined with first-principles calculations [22], demonstrating that F_4_-TCNQ within a Si NC solid film is not a shallow acceptor, but rather a deep acceptor with levels that can edge the conduction band minimum of the Si NC film. In fact, this dopant was used to increase the conductivity of the films by more than two orders of magnitude, although the majority carriers were electrons.

**III.2.3 Surface properties:** The NC surface is one of the most important variables in the engineering of its shape, intrinsic properties and stability in air and interaction with solvents and other substances. For nanoparticles with a clean surface or with a hydrogen-covered surface, the orientation dependence of the surface energy, as well as edge- and corner-energies (for less than 10^4^ atoms) determine the shape of the NCs grown under near-equilibrium quasi-static conditions. It has been found that diamond NCs are most stable in a truncated octahedral structure, but Si and Ge NCs are stable in a nearly-spherical geometry [61]. In any case, free standing Si and Ge NCs are usually grown in conditions very far from equilibrium, justifying the use of spherical models [62]. Diamond nanocrystals, however, have been grown in different shapes and configurations, and their optical properties have been found to be in discrepancy with theoretical models, presumably due to the contribution of defects [63].

As nanocrystals have a very large surface-to-volume ratio, their electronic and optical properties are largely determined by the surface or interface morphology. Amongst group-IV NCs, Si NCs are especially reactive in air, immediately forming an oxide cap. Surface oxidation also decreases the gap energy of the NC, shifting its absorption and photoluminescence edges to the red [64–65]. Models of Si NCs embedded in SiO_2_ also show an optical redshift following oxidation, and suggest that this effect is associated not only with the decreased quantum confinement, but also with the presence of silanone-like Si=O bonds [66]. As a consequence of the change in the electronic structure and dielectric screening, the ionization energy of dopants is also altered. The earliest oxidation stage corresponding to the formation of silanol surface groups was found to increase the electron binding energy of P, As and Sb, and to decrease the hole binding energy of B, Al, Ga and In [67].

Chlorine-covered Si NCs were found to have higher electron affinity, higher ionization energy and a lower optical absorption energy threshold than hydrogen-covered nanocrystals with the same size. Like the hydrogenated Si NCs, chlorinated Si NCs doped with phosphorus or boron require a high activation energy to transfer an electron or hole, respectively, to undoped Si NCs. The electronic levels of surface dangling bonds are similar for both types of surface passivation, although in the chlorinated Si NCs some fall off the narrower gap. Functionalisation with chlorine, nitrogen and fluorine was also found to be an effective way to control the bandgap of nanodiamonds [68].

Nanocrystals functionalized with organic groups have so far only been considered in a limited number of theoretical studies, for silicon [49,69–71] and diamond [72]. Functionalization with alkyl and aryl molecules is an important preparatory step for the conjugation of the surface to larger organic molecules. Regarding the direct influence on the physical properties, alkyl passivation was found to slightly change the optical gaps of silicon QDs, but to substantially decrease their ionization potentials and electron affinities and to affect their excited state properties. Nevertheless, there is still ample space for the modeling of the interaction of functionalized nanocrystals with solvents, polymers and biological molecules. A tight-binding parameterization has been proposed for this family of systems, offering a cheaper approach that may become a basis for a function-oriented treatment of organic-inorganic interfaces [48].

## Conclusion

Although group-IV NCs may emerge as a natural way to extend, enhance and control the properties of their respective bulk materials, the last two decades of research have shown that not only the device architecture, but also the growth, characterization and modeling techniques have to be redesigned to meet their very specific requirements.

Silicon and germanium NCs have revealed to be promising on the areas of optical luminescence, in which size-dependent luminescence and absorption of Si NCs is consistently reported. Memory devices are another promising area of application. So far, the greatest challenges continue to be the control of surface, interface and other deep levels, misfit strain, and doping efficiency.

From a theoretical point of view, even though one of the most important attributes of nanoparticles – their size-dependent optical gap – is a quantity hard to predict, modeling studies have been able to provide great insight into the fundamental optical and electronic properties of different types of NCs. An open issue still resides on the difficult task of bridging the length scales from the largest sizes achievable numerically to the smallest sizes produced experimentally.
